# Update: Influenza Activity in the United States During the 2016–17 Season and Composition of the 2017–18 Influenza Vaccine

**DOI:** 10.15585/mmwr.mm6625a3

**Published:** 2017-06-30

**Authors:** Lenee Blanton, Noreen Alabi, Desiree Mustaquim, Calli Taylor, Krista Kniss, Natalie Kramer, Alicia Budd, Shikha Garg, Charisse N. Cummings, Jessie Chung, Brendan Flannery, Alicia M. Fry, Wendy Sessions, Rebecca Garten, Xiyan Xu, Anwar Isa Abd Elal, Larisa Gubareva, John Barnes, Vivien Dugan, David E. Wentworth, Erin Burns, Jacqueline Katz, Daniel Jernigan, Lynnette Brammer

**Affiliations:** 1 Influenza Division, National Center for Immunization and Respiratory Diseases, CDC.

During the 2016–17 influenza season (October 2, 2016–May 20, 2017) in the United States, influenza activity* was moderate. Activity remained low through November, increased during December, and peaked in February nationally, although there were regional differences in the timing of influenza activity. Influenza A(H3N2) viruses predominated through mid-March and were predominant overall for the season, but influenza B viruses were most commonly reported from late March through May. This report summarizes influenza activity in the United States during October 2, 2016–May 20, 2017^†^ and updates the previous summary (*1*).

## Viral Surveillance

**FIGURE 1:**
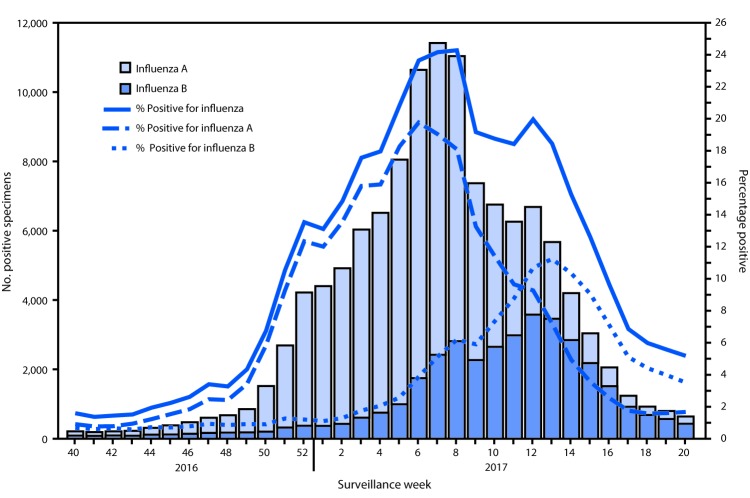
Number* and percentage of respiratory specimens testing positive for influenza reported by clinical laboratories, by influenza virus type and surveillance week — United States, October 2, 2016–May 20, 2017^†^ * Specimens from 121,223 (14.0%) of 865,168 persons tested positive during October 2, 2016–May 20, 2017. ^†^ As of June 9, 2017.

CDC receives influenza test results from public health and clinical laboratories located in all 50 states, Puerto Rico, and the District of Columbia through U.S. World Health Organization (WHO) Collaborating Laboratories and the National Respiratory and Enteric Virus Surveillance System. During October 2, 2016– May 20, 2017, clinical laboratories tested 865,168 specimens for influenza virus: 121,223 (14.0%) specimens tested positive for influenza virus (Figure 1), including 84,854 (70.0%) that tested positive for influenza A viruses and 36,369 (30.0%) that tested positive for influenza B viruses. Nationally, the percentage of specimens tested by clinical laboratories that were positive for influenza peaked during the 3 weeks ending February 11, February 18, and February 25, 2017 (weeks 6, 7, and 8) at 23.6%, 24.2%, and 24.3%, respectively. At a U.S. Department of Health and Human Services regional^§^ level, the timing of peak percent positivity varied. In regions 8 and 10, the percentage of viruses testing positive for influenza peaked during the week ending December 31, 2016 (week 52) and in region 9, the peak occurred during the week ending January 14, 2017 (week 2). In each of regions 1, 2, 3, 5, 6, and 7 the peak occurred from the week ending February 11 to February 25, 2017 (weeks 6–8). Region 4 also had peaks in influenza activity during the week ending February 11 through the week ending February 25, 2017 (weeks 6–8), and experienced a second peak during the week ending March 25, 2017 (week 12).

**FIGURE 2:**
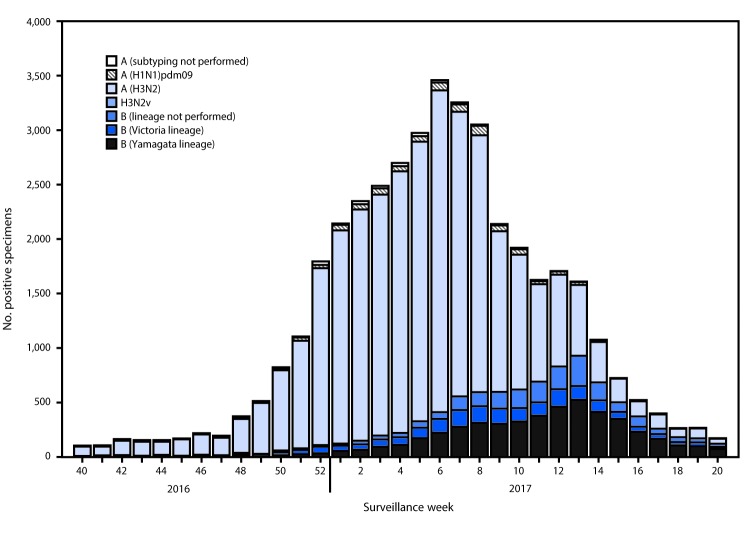
Number* of respiratory specimens testing positive for influenza reported by public health laboratories, by influenza virus type, subtype/lineage, and surveillance week — United States, October 2, 2016–May 20, 2017^†^ * N = 40,728. ^†^ As of June 9, 2017.

Public health laboratories tested a total of 84,303 specimens during October 2, 2016–May 20, 2017, and 40,728 were positive for influenza, including 31,736 (77.9%) influenza A and 8,992 (22.1%) influenza B viruses (Figure 2). Among the 31,411 influenza A viruses subtyped, 30,519 (97.2%) were influenza A(H3N2) viruses and 892 (2.8%) were influenza A(H1N1)pdm09 viruses. Influenza B lineage information was available for 6,875 (76.5%) influenza B viruses: 4,892 (71.2%) belonged to the B/Yamagata lineage and 1,983 (28.8%) to the B/Victoria lineage.

Age data were available for 36,426 of the influenza-positive patients tested by public health laboratories. Overall, 2,912 (8.0%) persons were aged 0–4 years, 11,066 (30.4%) were aged 5–24 years, 10,872 (29.8%) were aged 25–64 years, and 11,576 (31.8%) were aged ≥65 years. Influenza A(H3N2) viruses were predominant among all age groups, accounting for 70% of viruses identified among persons aged 0–4 years and 80% of viruses reported among persons aged ≥65 years. The largest proportion of reported influenza B viruses occurred in persons aged 5–24 years; influenza B viruses accounted for 28% of the viruses reported for that age group.

## Novel Influenza A Viruses

Three human infections with novel influenza A viruses were reported to CDC during the 2016–17 influenza season. The first was an infection with an influenza A(H1N2) variant (H1N2v) virus^¶^ reported by Iowa public health officials during the week ending November 19, 2016 (week 46). The patient was not hospitalized and fully recovered.

The second case, a human infection with a North American lineage avian influenza A(H7N2) virus, was reported to CDC during the week ending December 24, 2016 (week 51). The patient reported close, prolonged unprotected exposure to the respiratory secretions of infected, sick cats at a New York City animal shelter. This is the first avian influenza A(H7N2) virus infection in humans identified in the United States since 2003 and the first known human infection with an influenza A virus acquired through exposure to an ill cat. The patient was mildly ill, not hospitalized, and recovered completely.

The third case, an infection with an influenza A(H3N2) variant (H3N2v) virus, was detected through the Department of Defense Global Laboratory–based Influenza Surveillance Program and reported by Texas during the week ending April 29, 2017 (week 17). The patient reported contact with swine at an agricultural event the week preceding illness onset, was not hospitalized, and fully recovered.

## Antigenic and Genetic Characterization of Influenza Viruses

WHO collaborating laboratories in the United States are requested to submit a subset of influenza-positive respiratory specimens to CDC for further virus characterization. CDC characterizes influenza viruses through one or more laboratory tests, including the following: genomic sequencing, antigenic characterization by hemagglutination inhibition (HI), or neutralization assays. Historically, until vaccine effectiveness estimates are available,** HI data have been used most commonly to assess the antigenic similarity between vaccine reference viruses and circulating viruses to infer how well the vaccine might perform. Since the 2014–15 season, a substantial proportion of influenza A(H3N2) viruses have not yielded sufficient hemagglutination titers for antigenic characterization by HI assay. The focus reduction assay (a neutralization test), has been used to supplement HI testing for antigenic characterization of a subset of influenza A(H3N2) viruses. For nearly all influenza-positive surveillance samples received by CDC, next-generation sequencing^††^ is performed to determine the genetic identity of circulating viruses.

For the 2016–17 season, CDC genetically characterized 2,476 influenza viruses (311 influenza A(H1N1)pdm09, 1,280 influenza A(H3N2), and 885 influenza B viruses) collected by U.S. laboratories since October 1, 2016. The hemagglutinin (HA) gene of 309 (99%) of the 311 influenza A(H1N1)pdm09 viruses analyzed belonged to the predominant 6B.1 genetic subgroup, and the remaining two (1%) belonged to genetic group 6B. The HA gene of 1,187 (93%) influenza A(H3N2) viruses analyzed belonged to the 3C.2a genetic group, and the remaining 93 (7%) belonged to the 3C.3a genetic group. Of note, the 3C.2a genetic group includes an emerging subgroup known as 3C.2a1. The HA genes of 495 influenza B/Yamagata-lineage viruses analyzed all belonged to genetic group Y3. The HA genes of 390 influenza B/Victoria-lineage viruses all belonged to genetic group V1A. However, 78 (20%) of the 390 B/Victoria-lineage viruses have several amino acid changes, including two amino acid deletions at positions 162 and 163 in the HA gene, which alter the antigenic properties of these viruses. Viruses with these changes are currently being referred to as the “B/Victoria deletion variant subgroup.”

CDC has antigenically characterized 1,824 influenza viruses collected by U.S. laboratories since October 1, 2016 (296 influenza A(H1N1)pdm09, 772 influenza A(H3N2), and 756 influenza B viruses). Among the 296 A(H1N1)pdm09 viruses, 294 (99.3%) were antigenically characterized as A/California/7/2009-like, the influenza A(H1N1)pdm09 component of the 2016–17 Northern Hemisphere vaccine. Among the influenza A(H3N2) viruses, 730 (94.9%) were antigenically characterized as A/Hong Kong/4801/2014-like, a genetic group 3C.2a virus recommended as the A(H3N2) component of the 2016–17 Northern Hemisphere vaccine. Among 42 viruses that were antigenically different from A/Hong Kong/4801/2014-like viruses (i.e., reacted poorly with ferret antisera raised against reference viruses representing A/Hong Kong/4801/2014-like vaccine viruses), 36 (85.7%) belonged to genetic group 3C.3a, represented by the A/Switzerland/9715293/2013 reference virus, which was included as the A(H3N2) component of the 2015–16 Northern Hemisphere vaccine.

Among influenza B viruses, 327 B/Victoria-lineage viruses were antigenically characterized using postinfection ferret antisera and among these, 283 (86.5%) were antigenically characterized as B/Brisbane/60/2008-like, a recommended influenza B component of the 2016–17 Northern hemisphere trivalent and quadrivalent influenza vaccines. Among the 44 B/Victoria linage viruses that had reduced titers against B/Brisbane/60/2008-like viruses, 39 (88.6%) belong to the B/Victoria deletion variant subgroup. All 429 (100%) B/Yamagata-lineage viruses tested were antigenically characterized as B/Phuket/3073/2013-like, the recommended influenza B component of the 2016–17 Northern Hemisphere quadrivalent influenza vaccines.

## Antiviral Susceptibility of Influenza Viruses

CDC tested 2,569 influenza virus specimens (304 influenza A(H1N1)pdm09, 1,303 influenza A(H3N2), and 962 influenza B viruses) collected in the United States during October 1, 2016–May 20, 2017 for resistance to the influenza neuraminidase inhibitor antiviral medications oseltamivir, zanamivir, and peramivir, which are currently recommended for use against seasonal influenza. All 2,569 influenza viruses tested were found to be susceptible to all three of these antiviral medications. An additional 34 influenza A(H1N1)pdm09 viruses were tested for resistance to oseltamivir and peramivir and an additional 1,083 influenza A(H3N2) viruses were tested for resistance to oseltamivir and zanamivir; all were found to be susceptible to these antiviral medications.

## 2016–17 Influenza Vaccine Effectiveness

Data collected through the U.S. Influenza Vaccine Effectiveness Network during November 28, 2016–April 14, 2017, indicate that influenza vaccination this season reduced the overall risk for influenza-associated medical visits by 42% (95% CI = 35%–48%). Vaccine effectiveness against the predominant influenza A(H3N2) viruses was 34% (95% CI = 24%–42%) and vaccine effectiveness against influenza B viruses was 56% (95% CI = 47%–64%).

## Composition of the 2017–18 Influenza Vaccine

The Food and Drug Administration’s Vaccines and Related Biologic Products Advisory Committee (VRBPAC) has recommended that the 2017–18 influenza trivalent vaccine to be used in the United States contain an A/Michigan/45/2015 (H1N1)pdm09-like virus, an A/Hong Kong/4801/2014 (H3N2)-like virus, and a B/Brisbane/60/2008-like (B/Victoria lineage) virus. It is recommended that quadrivalent vaccines, which have two influenza B viruses, contain the viruses recommended for the trivalent vaccines, as well as a B/Phuket/3073/2013-like (B/Yamagata lineage) virus (*2*). This represents an update in the influenza A(H1N1) component compared with the composition of the 2016–17 influenza vaccines. The recommended Northern Hemisphere 2017–18 vaccine viruses are the same as the vaccine viruses recommended for inclusion in the 2017 Southern Hemisphere influenza vaccines. These vaccine recommendations were based on a number of factors, including global influenza virologic and epidemiologic surveillance, genetic and antigenic characterization, human serology studies, antiviral susceptibility, and the availability of candidate influenza viruses.

## Outpatient Illness Surveillance

**FIGURE 3:**
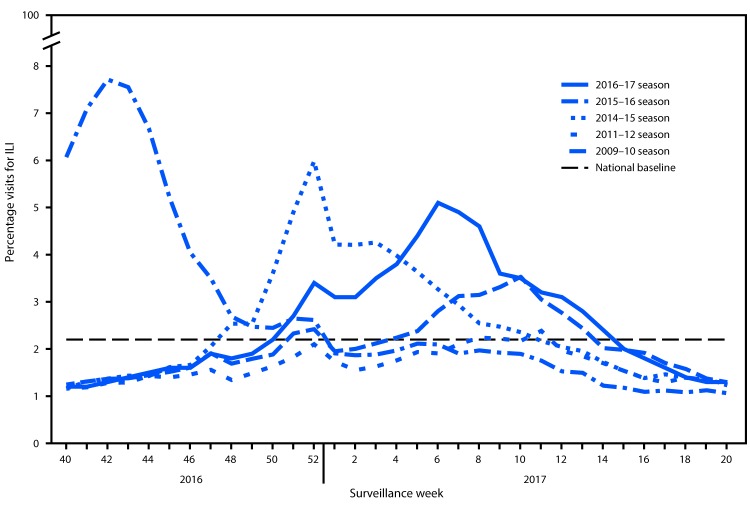
Percentage of visits for influenza-like illness (ILI)* reported to CDC, by surveillance week — Outpatient Influenza-Like Illness Surveillance Network, United States, 2016–17 influenza season and selected previous influenza seasons^†^ * Defined as fever (temperature ≥100.0°F [≥37.8°C], oral or equivalent) and cough and/or sore throat, without a known cause other than influenza. ^†^ As of June 9, 2017.

Nationally, the weekly percentage of outpatient visits for influenza-like illness^§§^ (ILI) to heath care providers participating in the U.S. Outpatient Influenza-like Illness Surveillance Network (ILINet) was at or above the national baseline^¶¶^ level of 2.2% from the week ending December 17, 2016 (week 50) and remained at or above baseline for 17 consecutive weeks during the 2016–17 season (Figure 3). Nationally, the peak percentage of outpatient visits for ILI was 5.1% and occurred during the week ending February 11, 2017 (week 6). During the 2011–12 through 2015–16 seasons, peak weekly percentages of outpatient visits for ILI ranged from 3.6% to 6.1% and remained at or above baseline levels for an average of 13 weeks (range = 1–20 weeks).

ILINet data are used to produce a weekly jurisdiction-level measure of ILI activity,*** ranging from minimal to high. The number of jurisdictions experiencing elevated ILI activity peaked during the week ending February 11, 2017 (week 6) when 31 states experienced high ILI activity. Thirty-seven jurisdictions (36 states and Puerto Rico) experienced high ILI activity during at least 1 week this season. The peak number of jurisdictions experiencing high ILI activity during a single week from 2011 to 2016 has ranged from four during the 2011–12 season to 45 during the 2014–15 season.

## Geographic Spread of Influenza Activity

State and territorial epidemiologists report the geographic distribution of influenza in their jurisdictions^†††^ through a weekly influenza activity code.^§§§^ The geographic distribution of influenza activity was most extensive during the week ending February 11, 2017 (week 6), when 47 jurisdictions reported widespread influenza activity. From 2011 to 2016, the peak number of jurisdictions reporting widespread influenza activity ranged from 20 during the 2011–12 season to 48 during the 2012–13 season.

## Influenza-Associated Hospitalizations

CDC monitors hospitalizations associated with laboratory-confirmed influenza infections in adults and children through the Influenza Hospitalization Surveillance Network (FluSurv-NET),^¶¶¶^ which covers approximately 27 million persons (9% of the U.S. population). During October 1, 2016–April 30, 2017, a total of 18,184 laboratory-confirmed influenza-related hospitalizations were reported, with a cumulative incidence for all age groups of 65.0 per 100,000 population. The hospitalization rate was highest among persons aged ≥65 years, who accounted for approximately 60% of reported influenza-associated hospitalizations.

The cumulative hospitalization rate during October 1, 2016–April 30, 2017, was 44.1 per 100,000 population among children aged 0–4 years, 16.7 among children and adolescents aged 5–17 years, 19.8 among adults aged 18–49 years, 65.1 among adults aged 50–64 years, and 290.5 among adults aged ≥65 years. Among all hospitalizations, 14,185 (78.0%) were associated with influenza A virus infections, 3,873 (21.2%) with influenza B virus infections, 62 (0.3%) with influenza A virus and influenza B virus co-infections, and 64 (0.4%) with an influenza virus for which the type was not determined. Among the 5,383 patients for which influenza A subtype information was available, 5,276 (98.0%) were infected with influenza A(H3N2) viruses and 107 (2.0%) were infected with influenza A(H1N1)pdm09 viruses.

Complete medical chart abstraction data were available for 7,315 (40.2%) hospitalized adults and children with laboratory-confirmed influenza as of June 9, 2017. Among 6,838 hospitalized adults with complete medical chart abstraction, 6,434 (94.1%) had at least one reported underlying medical condition that placed them at high risk**** for influenza-associated complications. The most commonly reported underlying medical conditions among adults were cardiovascular disease (51.8%), metabolic disorders (44.4%), obesity (34.8%), and chronic lung disease (30.1%). Among 477 hospitalized children with complete medical chart abstraction, 269 (56.4%) had at least one underlying medical condition; the most commonly reported of these were asthma (26.4%) and neurologic disorder (23.2%). Among 447 women of childbearing age (15–44 years) hospitalized with laboratory-confirmed influenza infections, 119 (26.6%) were pregnant.

## Pneumonia and Influenza-Associated Mortality

CDC tracks pneumonia and influenza (P&I)–attributed deaths through the National Center for Health Statistics (NCHS) Mortality Reporting System. The percentages of deaths attributed to P&I are released 2 weeks after the week of death to allow for collection of sufficient data to produce a stable P&I mortality percentage. Weekly mortality surveillance data include a combination of machine-coded and manually coded causes of death collected from death certificates. During the 2016–17 season, there was a backlog of data requiring manual coding within the NCHS mortality surveillance data. Work is underway to reduce and monitor the number of records awaiting manual coding. The percentages of deaths attributable to P&I are higher among manually coded records than the more rapidly available machine coded records and might result in initially reported P&I percentages that are lower than percentages calculated from final data.

During the 2016–17 season, based on data from NCHS, the proportion of deaths attributed to P&I was at or above the epidemic threshold^††††^ for 12 consecutive weeks from the week ending December 31, 2016 through the week ending March 18, 2017 (weeks 52–11). Mortality attributed to P&I peaked twice, once at 8.2% of all deaths during the week ending January 21, 2017 (week 3) and once at 8.1% during the week ending February 25, 2017 (week 8). During the 2011–12 through 2015–16 seasons, the peak weekly percentages of deaths attributable to P&I ranged from 8.7% during the 2011–12 season to 11.1% during the 2012–13 season.

## Influenza-Associated Pediatric Mortality

CDC monitors pediatric influenza deaths through the Influenza-Associated Pediatric Mortality Surveillance System. As of June 9, 2017, a total of 98 laboratory-confirmed influenza-associated pediatric deaths occurring during the 2016–17 season had been reported to CDC from Chicago, New York City, and 28 states. Of these 98 deaths, 46 were associated with an influenza A(H3N2) virus infection, three with an influenza A(H1N1)pdm09 virus infection, 14 with an influenza A virus for which no subtyping was performed, 34 with an influenza B virus infection, and one with an influenza virus for which the type was not determined. Since influenza-associated pediatric mortality became a nationally notifiable condition in 2004, the total number of influenza-associated pediatric deaths per season has ranged from 37 to 171, excluding the 2009 pandemic, during which 358 pediatric deaths were reported to CDC during April 15, 2009–October 2, 2010.

## Discussion

The 2016–17 influenza season was notable for the predominant circulation of influenza A(H3N2) viruses. Nationally, influenza activity peaked in mid-February, with influenza A(H3N2) viruses predominant early in the season through the week ending March 25, 2017 (week 12). Influenza B viruses became the predominant virus starting during week 13 (the week ending April 1, 2017) and remained the predominant virus through the end of May. The timing of peak influenza activity varied across the United States. Influenza activity peaked at least 1 month earlier (week 52 to week 2) in the western United States (regions 8, 9, and 10) than in the rest of the country. During the 2016–17 season, severity indicators (e.g., hospitalization and mortality rates) were within the range that has been observed during previous seasons when influenza A(H3N2) viruses predominated. Previous influenza A(H3N2)–predominant seasons have been associated with increased hospitalizations and deaths compared with seasons that were not influenza A(H3N2)–predominant, especially among children aged <5 years and adults aged ≥65 years (*3*,*4*). The majority of influenza viruses antigenically characterized at CDC were similar to the reference viruses representing the recommended components for the 2016–17 vaccine. A small subset of antigenically distinct influenza B/Victoria viruses was detected. No antiviral resistance to oseltamivir, zanamivir, or peramivir was identified among tested influenza viruses from the 2016–17 season.

Final vaccine effectiveness estimates of 34% (95% CI = 24%–42%) against illness caused by influenza A(H3N2) viruses and 56% (95% CI = 47%–64%) against illness caused by influenza B viruses were similar to previous seasons when recommended vaccine viruses have been well matched to (i.e.,“like”) circulating viruses, including the lower effectiveness observed against well-matched A(H3N2) viruses. Evidence of reduced protection against A(H3N2) viruses, even when vaccine viruses and circulating viruses are well matched, has been observed since the 2011–12 season. In general, vaccination with inactivated influenza vaccine has offered better protection against influenza A(H1N1) and influenza B viruses (*5*). Even during seasons when vaccine effectiveness is reduced, vaccination can offer substantial benefit and reduce the likelihood of severe outcomes such as hospitalization and death (*6*,*7*). During the 2012–13 season with estimated vaccination effectiveness against A(H3N2)-related illness of 39% (95% CI = 29%–47%), vaccination averted an estimated 5.6 million illnesses, 2.7 million medical visits, 61,500 hospitalizations, and 1,800 deaths (*8*). Estimates of the number of influenza illnesses, medical visits, and hospitalizations averted by influenza vaccination during the 2016–17 season are scheduled to be published in December 2017.

Influenza antiviral medications are an important adjunct to vaccination in the treatment and prevention of influenza. Treatment with influenza antiviral medications as close to the onset of illness as possible is recommended for patients with confirmed or suspected influenza who have severe, complicated, or progressive illness; who require hospitalization; or who are at high risk for influenza complications. Antiviral treatment should not be withheld from patients who are at high risk for complications or who are severely ill with suspected influenza infection, even if rapid antigen-detection influenza diagnostic test results are negative (*9*).

Although summer influenza activity in the United States typically is low, influenza cases and outbreaks have occurred during summer months and clinicians should remain vigilant in considering influenza in the differential diagnosis of summer respiratory illnesses. Testing for seasonal influenza viruses and monitoring for novel influenza A virus infections should continue year round. Health care providers also are reminded to consider novel influenza virus infections in persons with influenza-like illness and swine or poultry exposure, or with severe acute respiratory infection after travel to areas where avian influenza viruses have been detected. Providers should alert the local public health department if novel influenza virus infection is suspected. Clinical laboratories using a commercially available influenza diagnostic assay that includes influenza A virus subtype determination should contact their state public health laboratory to facilitate transport and additional testing of any specimen that is positive for influenza A, but for which the subtype cannot be determined. Public health laboratories should immediately send influenza A virus specimens that they cannot subtype using standard methods to CDC and submit all specimens that are otherwise unusual as soon as possible after identification.

Influenza surveillance reports for the United States are posted online weekly (https://www.cdc.gov/flu/weekly). Additional information regarding influenza viruses, influenza surveillance, influenza vaccine, influenza antiviral medications, and novel influenza A infections in humans is available online (https://www.cdc.gov/flu).

**Summary**

**What is already known about this topic?**

CDC collects, compiles, and analyzes data on influenza activity year round in the United States. Timing of influenza activity and predominant circulating influenza viruses vary by season.

**What is added by this report?**

During the 2016–17 influenza season, influenza activity remained low through November 2016, increased during December, and peaked in February. During October 2, 2016–May 20, 2017, influenza A(H3N2) viruses were identified most frequently, but influenza A(H1N1)pdm09 and influenza B viruses were also reported. Data collected from November 28, 2016 to April 14, 2017, indicate that influenza vaccination this season reduced the overall risk for influenza-associated medical visits by 42% (95% CI = 35%–48%). The composition of the 2017–18 influenza vaccine has been updated to better match circulating influenza viruses.

**What are the implications for public health practice?**

Annual influenza vaccination is recommended for all persons aged ≥6 months and remains the most effective way to prevent influenza illness. Antiviral medications are an important adjunct to vaccination in the treatment and prevention of influenza. Early treatment with neuraminidase inhibitor antiviral medications is recommended for patients with severe, complicated, or progressive influenza illness and those at higher risk for influenza complications, including adults aged ≥65 years.

## Footnotes

* The CDC influenza surveillance system collects five categories of information from eight data sources: 1) viral surveillance (U.S. World Health Organization collaborating laboratories, the National Respiratory and Enteric Virus Surveillance System, and novel influenza A virus case reporting); 2) outpatient illness surveillance (U.S. Outpatient Influenza-Like Illness Surveillance Network); 3) mortality (the National Center for Health Statistics Mortality Surveillance System and influenza-associated pediatric mortality reports); 4) hospitalizations (FluSurv-NET, which includes the Emerging Infections Program and surveillance in three additional states); and 5) summary of the geographic spread of influenza (state and territorial epidemiologist reports). https://www.cdc.gov/flu/weekly/fluactivitysurv.htm.

† Data as of June 9, 2017.

§ The 10 regions include the following jurisdictions: *Region 1*: Connecticut, Maine, Massachusetts, New Hampshire, Rhode Island, and Vermont; *Region 2*: New Jersey, New York, Puerto Rico, and the U.S. Virgin Islands; *Region 3*: Delaware, District of Columbia, Maryland, Pennsylvania, Virginia, and West Virginia; *Region 4*: Alabama, Florida, Georgia, Kentucky, Mississippi, North Carolina, South Carolina, and Tennessee; *Region 5*: Illinois, Indiana, Michigan, Minnesota, Ohio, and Wisconsin; *Region 6*: Arkansas, Louisiana, New Mexico, Oklahoma, and Texas; *Region 7*: Iowa, Kansas, Missouri, and Nebraska; *Region 8*: Colorado, Montana, North Dakota, South Dakota, Utah, and Wyoming; *Region 9*: Arizona, California, Hawaii, Nevada, American Samoa, Commonwealth of the Northern Mariana Islands, Federated States of Micronesia, Guam, Marshall Islands, and Republic of Palau; *Region 10*: Alaska, Idaho, Oregon, and Washington.

¶ Influenza viruses that circulate in swine are called swine influenza viruses when isolated from swine, but are called variant influenza viruses when isolated from humans. Seasonal influenza viruses that circulate worldwide in the human population have important antigenic and genetic differences from influenza viruses circulating in swine.

** A virus is considered “reference virus–like” if its hemagglutination inhibition (HI) or neutralization focus reduction assay (FRA) titer is within fourfold of the homologous HI/FRA titer of the reference strain. A virus is considered as low to the reference virus if there is an eightfold or greater reduction in the HI or FRA titer when compared with the homologous HI or FRA titer of the reference strain.

†† Next generation sequencing uses advanced molecular detection to identify gene sequences from each virus in a sample and thus reveals the genetic variations among many different influenza virus particles in a single sample; these methods also reveal the entire coding region of the genomes. https://www.cdc.gov/amd/project-summaries/influenza-vaccines.html.

§§ Defined as a fever (temperature ≥100.0°F [≥37.8°C], oral or equivalent) and cough and/or sore throat, without a known cause other than influenza.

¶¶ The national and regional baselines are the mean percentages of visits for influenza-like illness (ILI) during noninfluenza weeks for the previous three seasons plus two standard deviations. Noninfluenza weeks are defined as periods of ≥2 consecutive weeks during which each week accounted for <2% of the season’s total number of specimens that tested positive for influenza. National and regional percentages of patient visits for ILI are weighted based on state population. Use of the national baseline for regional data is not appropriate.

*** Activity levels are based on the percentage of outpatient visits in a jurisdiction attributed to ILI and are compared with the average percentage of ILI visits that occur during weeks with little or no influenza virus circulation. Activity levels range from minimal, corresponding to ILI activity from outpatient clinics at or below the average, to high, corresponding to ILI activity from outpatient clinics much higher than the average. Because the clinical definition of ILI is nonspecific, not all ILI is caused by influenza; however, when combined with laboratory data, the information on ILI activity provides a clearer picture of influenza activity in the United States.

¶¶¶ FluSurv-NET conducts population-based surveillance for laboratory-confirmed, influenza-associated hospitalizations in children and adolescents aged <18 years (since the 2003–04 influenza season) and adults aged ≥18 years (since the 2005–06 influenza season). The FluSurv-NET covers approximately 70 counties in the 10 Emerging Infections Program states (California, Colorado, Connecticut, Georgia, Maryland, Minnesota, New Mexico, New York, Oregon, and Tennessee) and additional Influenza Hospitalization Surveillance Project (IHSP) states. IHSP began during the 2009–10 season to enhance surveillance during the 2009 H1N1 pandemic. IHSP sites included Idaho, Iowa, Michigan, Oklahoma, and South Dakota during the 2009–10 season; Idaho, Michigan, Ohio, Oklahoma, Rhode Island, and Utah during the 2010–11 season; Michigan, Ohio, Rhode Island, and Utah during the 2011–12 season; Iowa, Michigan, Ohio, Rhode Island, and Utah during the 2012–13 season; and Michigan, Ohio, and Utah during the 2013–14, 2014–15, 2015–16, and 2016–17 seasons. Cumulative unadjusted incidence rates are calculated using CDC’s National Center for Health Statistics population estimates for the counties included in the surveillance catchment area. Laboratory confirmation is dependent on clinician-ordered influenza testing, and testing for influenza often is underutilized because of the poor reliability of rapid test results and greater reliance on clinical diagnosis for influenza. Therefore, cases identified as part of influenza hospitalization surveillance likely are an underestimation of the actual number of persons hospitalized with influenza.
